# Bacteriological Profile and Antibiogram of Cultures Isolated from Tracheal Secretions

**DOI:** 10.7759/cureus.4965

**Published:** 2019-06-21

**Authors:** Hassaan Ahmad, Abdullah Sadiq, Hamza Waqar Bhatti, Awais A Bhatti, Ahsan Tameez-ud-din, Ahmed Ibrahim, Noman A Chaudhary

**Affiliations:** 1 Miscellaneous, Rawalpindi Medical University, Rawalpindi, PAK; 2 Surgery, Rawalpindi Medical University, Rawalpindi, PAK; 3 Medicine, Rawalpindi Medical University, Rawalpindi, PAK; 4 Internal Medicine, Rawalpindi Medical University, Rawalpindi, PAK

**Keywords:** gram-negative bacteria, drug resistance, respiratory infections, tracheal secretions

## Abstract

Background

Respiratory infections are associated with high morbidity and mortality, especially in critically ill patients. The excessive use of broad-spectrum antibiotics has led to the development of drug resistance, thus resulting in the emergence of pathogens which are difficult to treat. The aim of this study was to identify common pathogens in tracheal secretions and to study the patterns of their sensitivity and resistance to various antibiotics.

Materials and methods

This descriptive cross-sectional study was conducted in the Department of Pathology and Microbiology, Holy Family Hospital, Rawalpindi, Pakistan, from August 2017 to December 2017, using the convenient sampling technique. Tracheal secretions from patients in the intensive care unit (ICU), tested in the Pathology and Microbiology Department of Holy Family Hospital, were included in the study. The culture was done on blood and MacConkey agar and the sensitivity pattern was performed on Muller Hinton agar. Data were analyzed using SPSS v.23.0.

Results

Out of the bacteria isolated from positive growth cultures, *Acinetobacter* (45; 53.6%) was the most common isolate followed by *Klebsiella *(11; 13.1%). *Acinetobacter* was most sensitive to tigecycline (94.7%), and gram-negative bacteria such as *Acinetobacter*, *Klebsiella,* and *Pseudomonas* showed resistance to higher generation cephalosporins.

Conclusion

*Acinetobacter* was the most common gram-negative bacilli isolated. Tigecycline was found to be effective against *Acinetobacter*.

## Introduction

​​​​​Tracheobronchial secretions are produced by mucous glands and goblet cells of the tracheobronchial tree [1]. These secretions are not only involved in the protection of the respiratory tract but are also responsible for the exchange of heat and water during breathing [2].

Respiratory infections are associated with high morbidity and mortality, especially in critically ill patients [3]. Such patients are usually maintained using invasive devices which themselves tend to be a major reservoir for hospital-acquired infections [4,5]. About 15% of hospital-acquired infections (HAIs) are caused by ventilator-associated pneumonia (VAP). This is the second-most-common HAI having the highest morbidity and mortality [6].

Moreover, the advent and increase in multi-drug resistant (MDR) pathogens serve as a major problem [7]. The excessive and indiscriminate use of broad-spectrum antibiotics has led to the development of these resilient microbes which are difficult to treat [8,9].

The aim of this study was to identify the common pathogens in tracheal secretions and to study the patterns of their sensitivity and resistance to various antibiotics, which can serve as guidelines to physicians for empirical treatment with proper antibiotics.

## Materials and methods

This descriptive cross-sectional study was conducted in the Department of Pathology and Microbiology, Holy Family Hospital, Rawalpindi, Pakistan, from August 2017 to December 2017. All patients whose tracheal secretion samples were tested in the Pathology and Microbiology Department of Holy Family Hospital were included in the study. In total, 186 samples were selected, regardless of age and gender, by using the convenient sampling technique. Tracheal secretions from patients admitted in the intensive care unit (ICU) for more than 48 hours were obtained by sterile suctioning using an endotracheal tube and suction catheter tip. Samples were inoculated on agar plates. The culture was done on blood and MacConkey agar and was incubated at 37°C for 24 to 48 hours. Microbes were identified under a microscope by observing morphological characteristics after gram staining and applying biochemical tests. Antibiotic sensitivity pattern was done on Muller Hinton agar using the Kirby Bauer disk diffusion method. Antibiotic discs containing amikacin, amoxicillin clavulanate (augmentin), aztreonam, ceftazidime, cefoperazone + sulbactam (sulzone), ciprofloxacin, ceftriaxone, cefepime, imipenem, tigecycline, piperacillin + tazobactam (tazocin), vancomycin, penicillin, gentamicin, linezolid, chloramphenicol, erythromycin, methicillin, ampicillin, and moxifloxacin were obtained and used as per the manufacturer's instructions.

Data were entered and analyzed using the Statistical Package for Social Sciences (SPSS) v.23.0 (IBM, Armonk, US). Descriptive statistics were applied to find frequencies and percentages. Charts and tables were constructed.

## Results

A total of 186 samples were collected during this study period with 85 (45.7%) samples from males, 95 (51.1%) from females, while gender was missing for six (3.2%) patients. Positive growth was observed in 84 (45.2%) samples. The gram-negative bacilli contributed a major number of isolates (75; 68.4%), the remaining nine (31.6%) were caused by gram-positive cocci.

**Figure 1:**
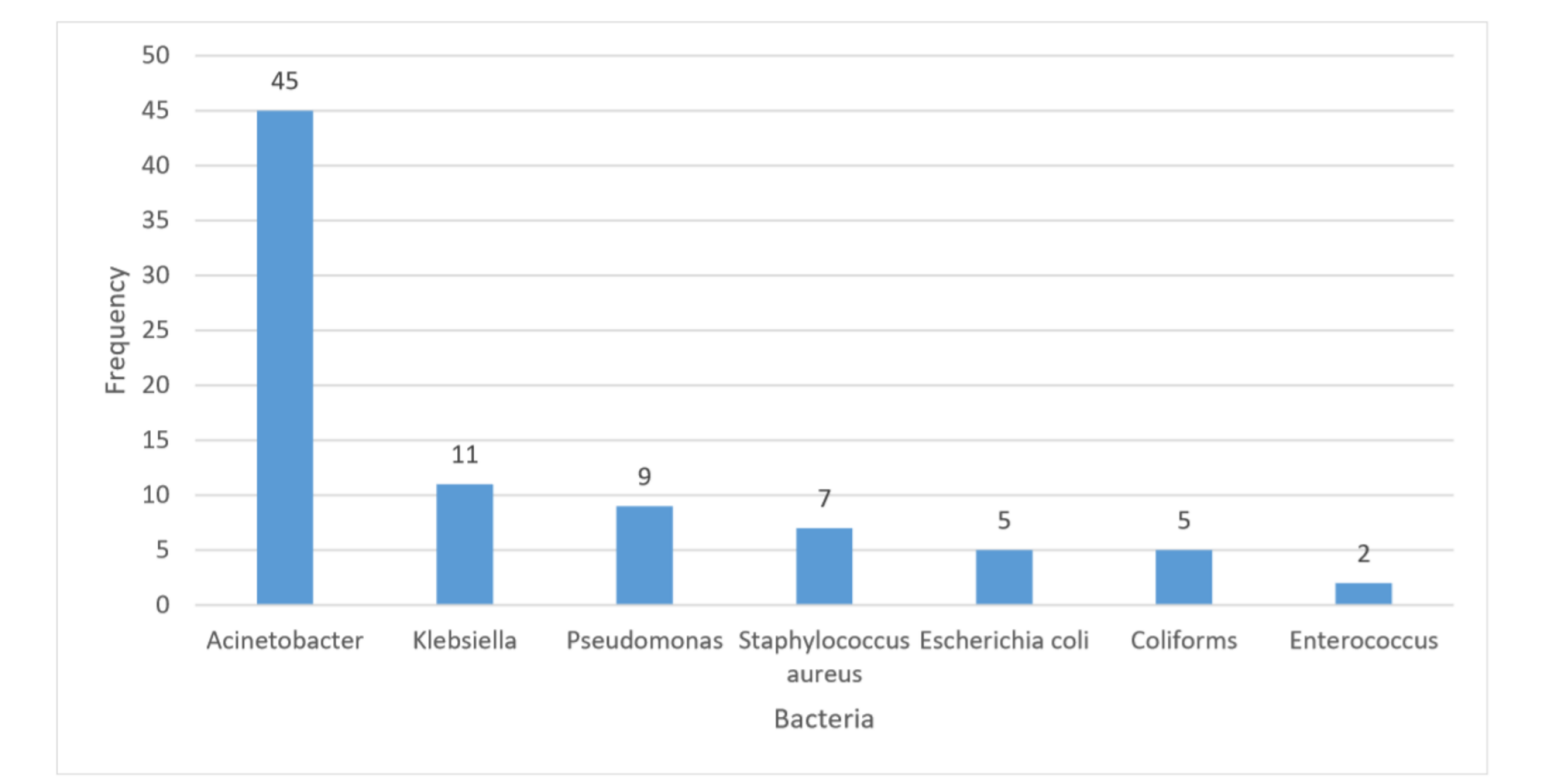
Bacterial growth in tracheal secretions (n=84)

Out of the bacteria isolated from positive growth cultures, *Acinetobacter* (45; 53.6%) was the most common isolate followed by *Klebsiella* (11; 13.1%). There were seven cases (8.3%) of *Staphylococcus aureus* which included one (1.3%) methicillin-resistant *Staphylococcus aureus* (MRSA). The least common isolate was *Enterococcus* (2; 2.4%) (Figure 1).

*Acinetobacter* was most sensitive to tigecycline (94.7%) while the gram-positive cocci, i.e., *Staphylococcus aureus* and *Enterococcus* showed susceptibility to vancomycin (100%) (Table 1).

**Table 1:** Antibiotic sensitivity pattern of gram-positive cocci in tracheal secretions

Antibiotics	Staphylococcus aureus	Enterococcus
Vancomycin	100%	100%
Penicillin	0%	-
Gentamicin	100%	0%
Linezolid	-	100%
Chloramphenicol	100%	-
Erythromycin	25%	0%
Methicillin	0%	-
Ampicillin	-	0%
Moxifloxacin	-	50%

The gram-negative bacilli such as *Acinetobacter, Klebsiella*, and *Pseudomonas* showed resistance to higher generation cephalosporins such as ceftriaxone and cefepime (100%). The susceptibility pattern of gram-negative bacilli is shown in Table 2.

**Table 2:** Antibiotic sensitivity pattern of gram-negative bacilli in tracheal secretions

Antibiotic	Acinetobacter	Klebsiella	Pseudomonas	Escherichia coli	Coliforms
Amikacin	4.7%	54.5%	22.2%	80%	80%
Amoxicilllin clavulanate (augmentin)	0%	0%	-	0%	40%
Aztreonam	-	-	28.6%	-	-
Ceftazidime	0%	0%	20%	0%	-
Cefoperazone + sulbactam (sulzone)	7.7%	81.8%	66.7%	50%	60%
Ciprofloxacin	0%	0%	22.2%	20%	50%
Ceftriaxone	0%	0%	0%	25%	60%
Cefepime	0%	0%	0%	33.3%	50%
Imipenem	2.5%	70%	28.6%	100%	40%
Tigecycline	94.7%	-	-	-	-
Piperacillin + tazobactam (tazocin)	2.5%	71.4%	12.5%	50%	75%

## Discussion

The resistance to conventional antibiotics is severely increasing in bacteria in clinical and non-clinical settings [10]. The rate of nosocomial infection is also steadily mounting in the patients admitted in the ICU due to excessive invasive procedures performed including artificial ventilator support [11]. This constantly emerging resistance is a serious situation implying the need for new regulations for the cautious use of antibiotics and refining the conditions of hospitals to prevent further exacerbation of resistance shown by the bacteria.

The percentage of samples showing positive growth in our study was 45.2%. In a study conducted by Gupta et al., the percentage of positive growth was 53% [7]. In another study by Chandra et al., the positive samples were 72.3% [12]. In a study conducted in the setting of Pakistan by Malik et al., the positive cultures came out to be 83% [13]. This marked decrease in our study can be attributed to the better infection control measures in the ICU setup of our hospital. However, the convenient sampling technique used in our study might be a limiting factor for the decreased percentage of positive growth.

In our study, gram-negative bacilli were more common causative agents (68.4%) as compared to gram-positive cocci, which were 31.6% of the total positive cultures. This was consistent with other researches by Chandra et al., in which the gram-negative bacilli were 85% and Gupta et al., in which 86% of the samples were gram-negative bacilli [7,12]. A study by Deepti et al. showed that more of the isolates from the patients were gram-negative enteric aerobic bacteria, with *Klebsiella* being the most common species followed by *Acinetobacter* and *Pseudomonas* [12]. This can be attributed to the fact that the majority of the nosocomial infections are caused by gram-negative bacteria which are more dangerous and difficult to treat. This calls for strict measures against the spread of gram-negative bacilli, especially in the ICU setting.

In our study, *Acinetobacter* (53.6%) was the most common isolate. In a study by Malik et al., conducted in Lahore, Pakistan, the commonest bacterium isolated from tracheal secretions was *Klebsiella pneumoniae* (35.4%) [13]. Similarly, in a study by Chandra et al., *Klebsiella* (32.35%) was the most common isolate [12]. However, in one study by George et al., *Acinetobacter* was the most common isolate (37.5%), followed by *Pseudomonas* (21.8%) and *Klebsiella* (15.6%) [14]. The rise in *Acinetobacter* in our study, especially in the ICU setup, can be attributed to the dramatic increase in the occurrence of multi-drug resistant isolates. In addition, this organism has the ability to survive in humid and dry conditions for longer periods, resulting in nosocomial outbreaks [15,16].

The second most common isolate in our study was *Klebsiella* (13.1%). In a study conducted by Malik et al., in Pakistan, the most common bacterium isolated from tracheal secretions was *Klebsiella pneumoniae* (35.4%) [13]. Another study by Chandra et al. showed *Klebsiella* (32.35%) to be the most common isolate [12]. *Pseudomonas* was the third-most common isolate present in our study. A study by Chandra et al. showed similar results [12].

In our study, *Acinetobacter* was most sensitive to tigecycline (94.7%) followed by sulzone (7.7%). A study by Malik et al. showed 69% sensitivity of *Acinetobacter* to sulzone [17]. In another study by Anusha et al., *Acinetobacter* was most sensitive to imipenem and ciprofloxacin [3]. The decrease in sensitivity, especially to sulzone, can be attributed to the emergence of resistance to the drugs that were conventionally administered.

Our study showed *Klebsiella* to be most sensitive to sulzone (81.8%) and tazocin (71.4%). *Pseudomonas* was shown to be 66.7% sensitive to sulzone. In a study by Malik et al., the sensitivity of *Klebsiella* to sulzone was 62%, while that of *Pseudomonas* was 71.2% [17]. Both *Klebsiella* and *Pseudomonas* showed a gradual decrease in the sensitivity to drugs. These gram-negative bacteria have developed resistance to multiple drugs which can be associated with cross-infections and other factors like the unjust use of antibiotics [18].

The other gram-negative bacilli of our study included *Escherichia coli* and coliforms which were sensitive to amikacin (80%) and imipenem (100%). *Escherichia coli*, in a study by Anusha et al., showed sensitivity to both amikacin and imipenem [3].

The gram-positive cocci, *Staphylococcus aureus* and *Enterococcus,* were sensitive to vancomycin (100%). This was consistent with a study by Gupta et al. which showed no change in antimicrobial resistance patterns [7].

The resistance patterns of bacteria in our study showed *Acinetobacter* to be 100% resistant to ceftazidime, ceftriaxone, ciprofloxacin, and cefepime. In the research by Gupta et al., *Acinetobacter* showed resistance to cephalosporins and aminoglycosides [7]. In another research by Chandra et al., 86% of the samples showed resistance against ceftriaxone and ceftazidime, each [12]. This depicted a pattern of increasing resistance to the drugs. In our study, out of all the gram-negative bacilli, *Acinetobacter* showed increasing resistance to carbapenems. Another study in Pakistan by Malik et al. showed similar results [17]. This is an alarming situation as the emergence of multi-drug resistant (MDR) and extensive drug resistant (XDR) pathogens in tracheal secretions is increasing morbidity and mortality in patients, making treatment difficult and expensive.

The remaining gram-negative bacteria in our study displayed resistance to the higher generations of cephalosporins, fluoroquinolones, penicillin, and drugs such as ceftazidime, ceftriaxone, ciprofloxacin, and cefepime. The broad-spectrum antibiotics showed sensitivity to an extent, but there was an alarming rise in the resistance to drugs such as carbapenems. This was in consistency with the pattern shown in the research by Gupta et al. and Malik et al. [7,17]

The limitation of our research included the decreased time duration of the study. Moreover, the study was carried out in a single tertiary care hospital due to which the generalization of results to the whole population cannot be carried out.

## Conclusions

Gram-negative bacilli were predominant in tracheal secretions with *Acinetobacter* being the most common species isolated. Tigecycline was found to be effective against *Acinetobacter*. The resistance to cephalosporins and penicillins is shown to be established by *Acinetobacter, Pseudomonas,* and *Klebsiella*. One of the grave concerns related to hospital-acquired infections is the upsurge of multi-drug resistance among the respiratory pathogens which have also extended into the community. There is a need for limited and objective use of antibiotic therapy according to the new guidelines modified under the light of such researches.
